# News and Notes

**Published:** 1905-02

**Authors:** 


### NEWS AND NOTES.

---

THE marriage is announced of Dr. W. A. Starnes and Miss Tor bett.

---

DR. G. P. HUGULEY has been appointed on the staff of the King's Daughters' Hospital.

---

DR. BERNARD WOLFF has been elected a member of the Board of Health of the city of Atlanta.

---

DR. F. W. McRAE has returned from a vacation in South Georgia and has quite recovered his health.

---

THE Georgia State Board of Health met at the State Capitol on January 6. The result of the proceedings has not been given out.

---

DR. J. N. ELLIS has been elected attending surgeon to the Grady Hospital to fill the vacancy caused by resignation of Dr. H. P. Cooper.

---

THE several bodies concerned in the anti-tuberculous movement will meet in Atlanta at the same time as the annual meeting of the State Medical Association, April 17-19.

---

DR. FRANK M. RIDLEY of LaGrange, and Dr. W. D. Travis of Covington, have been appointed on the State Board of Medical Examiners to serve three years from January 7th.

THE Prussian Minister of the Interior of Berlin, has reissued the old decree forbidding public exhibitions of hypnotism on the ground that such proceedings are injurious to the subjects hypnotized.

---

A HOSPITAL, to cost $75,000, is to be erected in Chicago, the physicians of which must use no alcohol in their prescriptions. The hospital will be called the Francis E. Willard National Temperance Hospital. The three schools of practice—regular, homeopathic, and eclectic—will be represented on the medical staff.

---

THE Civil Service Commission has decided that hereafter persons suffering from consumption will not be employed in the post-office or in any other government position from which they are likely to spread the disease.

---

THE annual election of officers of the Atlanta Society of Medicine resulted in the reelection of the old officers, the only new addition being that of Dr. Cyrus W. Strickler to the Board of Censors.

This is a highly merited compliment to Dr. Alex. W. Stirling. Dr. Stirling has made one of the most efficient, active and energetic presidents in the history of the society, and his reelection to the highest office in it not only reflects the esteem of its members in a notable degree but insures a continuance of the flourishing condition in which the society has been placed very largely through Dr. Stirling's efforts.

A program for the ensuing year is appended.

